# Colorimetric and spectrophotometric measurements of orthodontic thermoplastic aligners exposed to various staining sources and cleaning methods

**DOI:** 10.1186/s13005-020-00218-2

**Published:** 2020-02-18

**Authors:** Gabrielle Bernard, Pierre Rompré, Jason Robert Tavares, Andrée Montpetit

**Affiliations:** 1 Department of Oral Health – Orthodontics Section, Faculty of Dental Medicine, Université de Montréal, Québec, Canada; 2 CREPEC, Department of Chemical Engineering, Polytechnique Montréal, P.O. Box 6079, Stat. Centre-Ville, Montréal, Québec H3C 3A7 Canada

**Keywords:** Aligner, Orthodontics, Colorimetry, Staining, Cleaning

## Abstract

**Background:**

Manufacturers of orthodontic aligners suggest that users remove appliances every time they consume solid foods or any drink (except water). This is to avoid a color change within the clear thermoplastic material of which they are made. However, limited quantitative evidence exists to guide users and practitioners in this regard. Herein, we evaluated the color stability of the polymer forming three different American brands of aligners and the stain-removal potential of two cleansers to provide such guidelines.

**Methods:**

The removable appliances (300 specimens, 100 per brand) were exposed to different staining agents common in a regular diet (coffee, black tea, red wine, cola) or to a control solution in vitro over 12 h or 7 days. The three brands evaluated were Invisalign®, ClearCorrect® and Minor Tooth Movement®. These were then cleaned by using either Invisalign® cleaning crystals or the Cordless Sonic Cleaner combined with a Retainer Brite® tablet. The CIELAB color space approach was used to compare color changes (ΔE) in aligners before immersion (T0), after a 12-h exposure (T1), after a 7-day exposure (T2) and after cleaning (T3). Statistical methods (Levene’s test, ANOVA, Brunner-Langer model, Tukey’s range test and t-test) were used to identify interactions between the brands themselves or between the brands and the cleaning methods. Statistical analyses were performed at the .05 significance level.

**Results:**

A 12-h or 7-day exposure to instant coffee or red wine significantly colored the Invisalign® aligners compared to the two other brands. Black tea created an important extrinsic color change for all three brands after 7 days. Clinically, both cleaning methods showed a better efficacy in removing stains from black tea compared to other staining agents.

**Conclusions:**

The Invisalign® aligners were more prone to pigmentation than the ClearCorrect® or the Minor Tooth Movement® devices after an exposure to coffee or red wine. Black tea caused important stains on the surface of the three tested brands. Both cleansing methods performed similarly.

## Background

Thermoplastic orthodontic aligners are a popular replacement option to conventional fixed appliances such as braces [1]. These removable appliances are sought, in particular, by adults looking for a more aesthetic option with less metal exposure [2]. Patients are normally asked to wear their aligners full-time except when they eat or drink anything except water, or when they brush or floss their teeth [3, 4]. However, many patients do not have complete compliance [5] and consume coloring agents with their devices despite the orthodontists’ or manufacturers’ recommendations [6]. This leads to a change within the polymer forming the aligners, affecting their transparency, which is one of their main advantages [7–9].

Few studies have examined the color stability of orthodontic aligners to staining agents, and only on a limited set of brands available on the market [7–9]. While there have been studies evaluating the removal of bacterial biofilms at the surface of aligners by different cleaning products and methods [6, 10], only a select few articles compared the transparency of aligners after cleaning [11–13]. To our knowledge, no study exists in which thermoplastic aligners are both exposed to coloring agents and subjected to a cleansing cycle to verify the color changes of the devices.

The main objective of our study was to evaluate the stain resistance of three different American aligner brands for up to 7 days in a staining solution in vitro. Our second aim was to evaluate the stain-removal potential of two cleaning techniques after immersion.

## Methods

Three hundred aligners were used for testing: 100 were from Invisalign® (INV) (Align Technology Inc., San Jose, CA, USA), 100 from ClearCorrect® (CC) (ClearCorrect LLC, Rock Round, TX, USA) and 100 from Minor Tooth Movement® (MTM) (Dentsply Sirona Inc., York, PA, USA). The vast majority of aligners were not identical in that they were not necessarily all thermo-formed on the same model.

INV appliances are made from SmartTrack, a multilayer thermoplastic polyurethane combined with an integrated elastomer [8, 14]. CC devices are made of Zendura®, a polyurethane resin [15]. The MTM Safety Data Sheet states it is composed of Essix Ace, a polymer combining a copolyester (95%) and trade secret material (5%) [16]. The copolyester was previously identified via Fourier transform infrared spectroscopy (FTIR) as polyethylene terephthalate glycol-modified (PETG) [17].

**Fig. 1:**
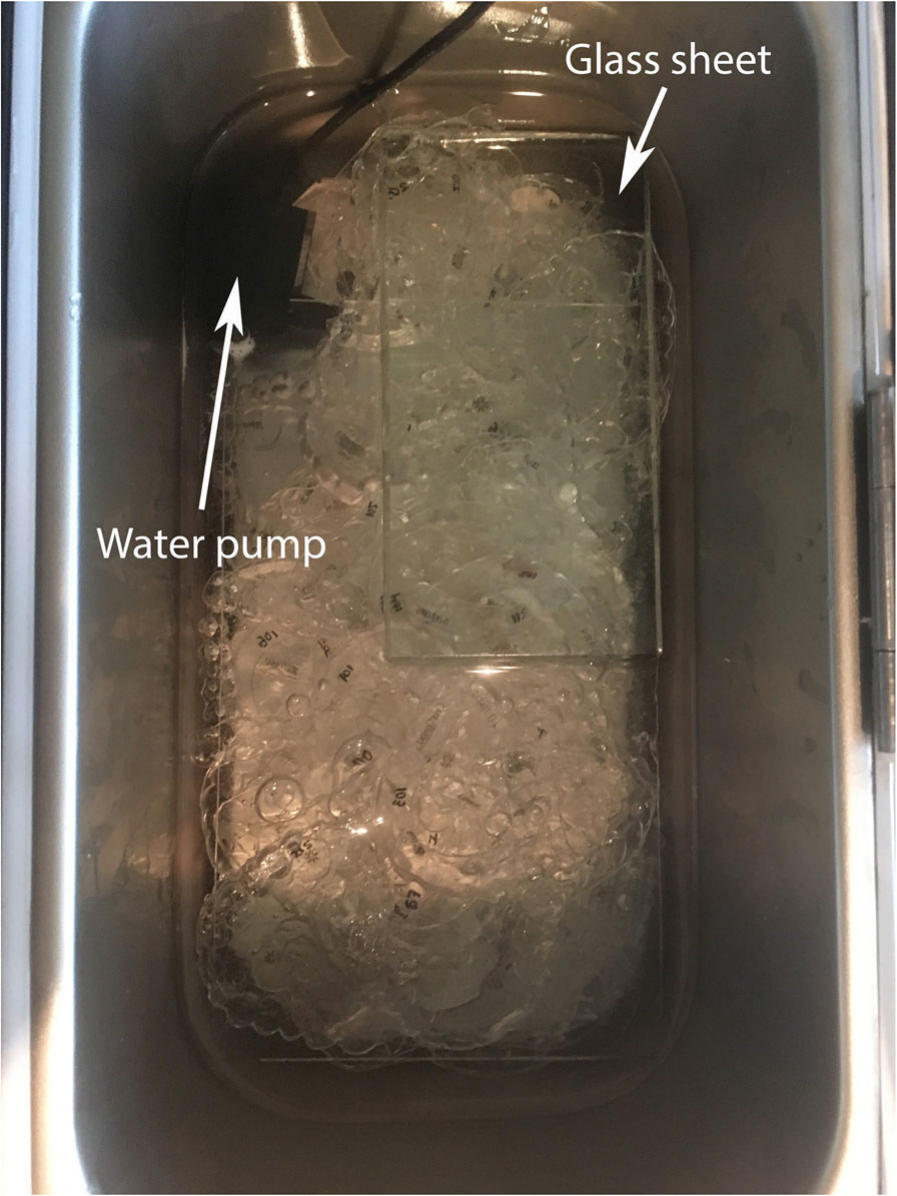
Specimens submerged into the control solution with two glass sheets and the Gold Wing water pump

Five different coloring media were employed. Each had a volume of 2.5 L and was maintained at 37 ± 1 °C in a thermostated water bath (PolyScience WB05A11B, PolyScience, Niles, IL, USA). Fresh solutions were prepared daily for the 7-day immersions. A submersible water pump (Gold Wing 3,5 V–9 V 3 W USB-1020, Goldwing, Beijing, China) was used during the immersions to ensure mixing, and two glass sheets kept the specimens submerged (Fig. 1). The instant coffee solution consisted of 30 g of instant coffee powder (Nescafé® Original, Nestlé, Vevey, Vaud, Switzerland) per 2.5 L of boiling distilled water (as per a previous study [9]). For the tea (English Breakfast Tea, Twinings, Andover, England), 9 bags per 2.5 L of boiling distilled water was used (steeped for 4 minutes). The cola (Coca-Cola, Coca-Cola Company, Atlanta, GA, USA) and red wine (Merlot/Malbec Astica, Bodegas Trapiche, Mendoza, Argentina) coloring media were used as supplied. For the control solution, 500 mL of saliva replacement gel (Biotène® Oral Balance, GlaxoSmithKline, Brentfort, England) diluted in 2 L of distilled water was employed (as per [8]). The 100 appliances per brand were divided in five groups of 20 specimens, each subgroup being exposed to either one of the four coloring media or the control solution. Then, each group of 20 aligners per solution was split again into two groups of 10 shells, which were either immersed in the substrate over 12 h or 7 days. Finally, the 10 specimens exposed to the same substrate (and for the same time) were separated into two subgroups of 5 appliances in order to be eventually cleansed by one of two methods. An additional chart flow schematic illustrates the sample distribution (see Additional file 1). Aligners were briefly immersed in distilled water after a 12-h exposure before analysis. For a 7-day immersion, aligners were quickly rinsed with distilled water every 24 h before being re-immersed in a fresh solution bath.

Among the various products on the market, two cleaning methods were retained: Invisalign® cleaning crystals (Align Technology Inc., San Jose, CA, USA) and the Cordless Sonic Cleaner combined with a Retainer Brite® tablet (Dentsply Sirona Inc., York, PA, USA). These two techniques were chosen as they constitute cleaning options offered by two of the three American companies producing the aligners studied in our research. Aligners were cleaned separately with each approach lasting 15 min. One bag of crystals was diluted in 100 mL of distilled water at room temperature (22 °C) immediately before aligner immersion. In the case of the Retainer Brite® tablet, it was incorporated in an active Cordless Sonic Cleaner bath containing 100 mL of distilled water at room temperature (22 °C) at the same time as the aligner to be cleaned. Before color analysis, each sample was rinsed with distilled water and dried with compressed air.

### Colorimetry

The color changes (ΔE) were calculated via the Commission Internationale de I’Eclairage (CIE) L*a*b* color system. L* indicates luminosity from darkness to lightness (values from 0 to 100, 0 = black and 100 = white). a* and b* are two axes of the chromatic scale. A positive a* corresponds to red, while negative means green. A positive b* corresponds to yellow whereas negative is blue [18]. *ΔE* was calculated in accordance with the formula [7, 18]:

$$ \varDelta \kern0.1em E={\left({\left(\varDelta \kern0.1em L\ast \right)}^2+{\left(\varDelta \kern0.1em a\ast \right)}^2+{\left(\varDelta \kern0.1em b\ast \right)}^2\right)}^{1/2} $$

ΔL*, Δa* and Δb* are the subtractions of the L*, a* and b* color parameters collected at various times T1 − T0, T2 − T0, T3-T1, T3-T2 and T3-T0 (for 12 h and 7d separately):

- T0: before specimen immersion (as-received aligner)
- T1: after a twelve-hour exposure to a staining solution
- T2: after a seven-day exposure to a staining solution
- T3: after a 15-min cleaning by one of the two techniques

To obtain the color parameters, the 300 aligners were scanned at T0, T1 or T2 and T3 with an Epson Perfection V700 Photo flatbed scanner (Seiko Epson Corporation, Suwa, Nagano, Japan). Before every measurement session, the scanner was calibrated with an IT8 SilverFast Fuji transparent target (LaserSoft Imaging, Kiel, Germany) and the SilverFast Ai Studio 8 one software (LaserSoft Imaging, Kiel, Germany). The positive and transparent film scanned images had a resolution of 1800 ppi. They were saved as uncompressed TIFF images to retain all data.

The images were analyzed with Adobe Photoshop® CS6 software (Adobe, San Jose, CA, USA). Five regions were kept as similar as possible from one measurement session to the other in order to obtain a mean as repeatable as possible. The chosen areas were situated in the posterior part of each arch in order to avoid any overlap in the polymer. The tips of the cuspids, the grooves or the pits of the teeth were favorably selected. The National Bureau of Standards (NBS) system was used to offer a clinical interpretation (perception) to the color change values (ΔE) obtained [7–18] (Table 1):

$$ NBS=\varDelta \kern0.1em E\times 0.92 $$

**Table 1:** National bureau of standards ratings

National Bureau of Standards units	Description of color change
0.0–0.5	Trace: extremely slight change
0.5–1.5	Slight: slight change
1.5–3.0	Noticeable: perceivable
3.0–6.0	Appreciable: marked change
6.0–12.0	Much: extremely marked change
12.0 and more	Very much: change to other color

To our knowledge, this method to assess color change of aligners by scanning has not been described before. The only other instance we have identified describing this approach is from a thesis studying orthodontic elastomeric auxiliaries [19]. We believe that the use of a cursor to situate a specific region of a magnified aligner at high resolution is more precise than the use of a larger tip intra-oral colorimeter.

Statistical analyses were conducted with the IBM *SPSS Statistics* 25.0 and the *Statistical Analysis System* (SAS) 9.4 software packages. Levene’s test was used to assess homogeneity of variances across groups. One-way ANOVA and nonparametric ANOVA-type statistics (Brunner-Langer [20]) were used when appropriate to compare the mean color changes among the 3 brands. Two-way ANOVA or nonparametric ANOVA-type statistics (Brunner-Langer) identified interactions between the brands and the cleaning techniques concerning the color changes. Tukey correction was applied for pairwise comparisons. T-tests were used to compare the effects of different cleaning methods, if there was an interaction between a brand and the two cleaning techniques. A *p* value < 0.05 was considered statistically significant. When a significant interaction effect was found, only the highest level of interaction was reported. Lower level significant interactions were not mentioned in this article if they were not visible by the human eye or only appreciable by a skilled individual.

### Spectrophotometry

Fifteen additional aligners (5 per brand) were analyzed via FTIR spectrophotometry to identify the polymer composition of the internal and external surfaces of the different shells. A Thermo Fisher Nicolet iS5 (Thermo Fisher Scientific, Waltham, MA, USA) in Attenuated Total Reflectance (ATR) mode (iD7 with a diamond plate accessory) was used in conjunction with the OMNIC FTIR 9.2.86 software. Three different regions per surface were sampled. The scan resolution was 4 cm^− 1^ and the scan range was 400 to 4000 cm^− 1^. Sixteen scans were averaged by the software.

## Results

The 100 MTM aligners used were formed out of one of two different models (one per dental arch) used by Dentsply Sirona© (the company that supplied them for our research). The CC and INV aligners were all different models. It was relevant for us to know whether comparing sets of five aligners formed on different models and, consequently, changing the location of the five chosen areas per shell from one appliance to the other affected the results. To that end, we compared the standard deviations obtained for readings on MTM aligners compared to CC ones and found no statistical difference (*p* = 0.504).

**Fig. 2:**
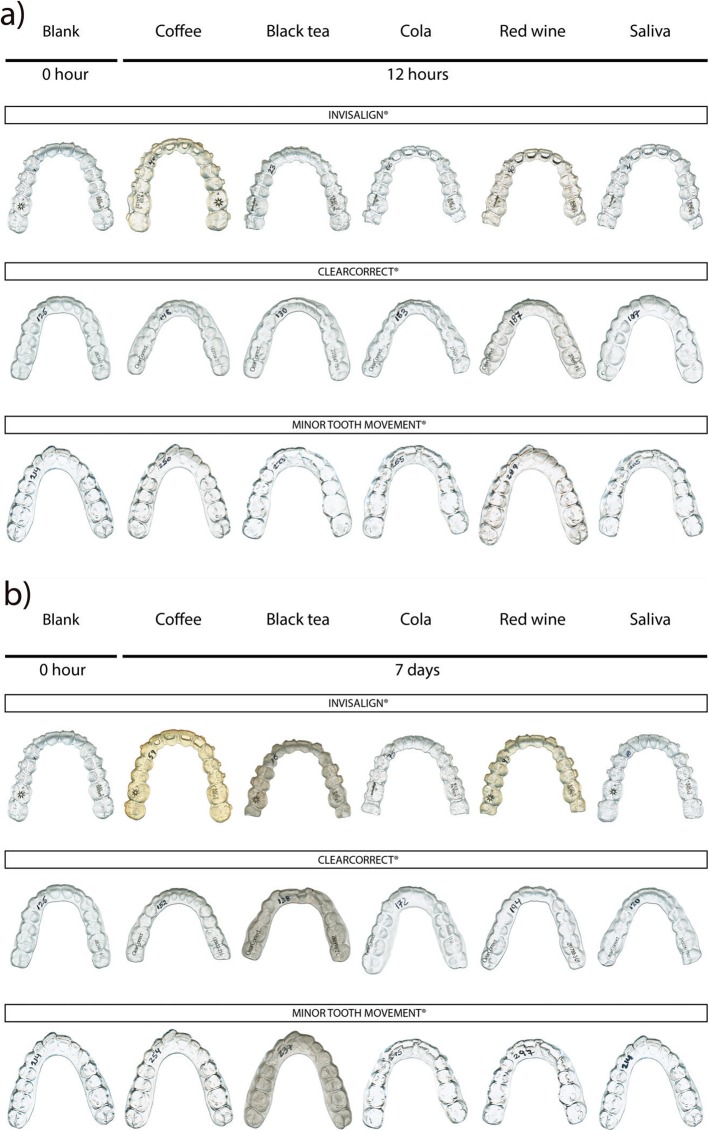
Photographs of the three brands of aligners before and after staining in each solution (**a**) for 12 h (**b**) for 7 days

All colorimetric results are listed in Table 2. Color change after a 12-h exposure showed a significant difference in mean values for INV compared to the two other brands for coffee (Brunner-Langer, *p* < 0.0001 for INV-CC and INV-MTM) and red wine (one-way ANOVA, *p* < 0.001 for INV-CC and INV-MTM) (Fig. 2).

**Table 2:** Means (±SD) of color changes (∆E) at various time intervals for 3 aligners brands, 5 staining agents and 2 cleansers

Brand	Staining agent	Cleanser	∆E (T1-T0) IC	∆E (T2-T0) IC	∆E (T3-T1)	IC	∆E (T3-T2)	IC	∆E (T3-T0) 12 h group	IC	∆E (T3-T0) 7d group	IC
INV	Coffee	Crystals	7.12 (±2.27) a	27.76 (±4.99) a	1.51 (±0.38)	a	1.72 (±1.23)	a	6.29 (±2.35)	a	29.83 (±2.50)	a
		RB			1.41 (±0.59)		1.47 (±0.57)		5.48 (±2.77)		22.94 (±3.20)	
	Cola	Crystals	0.92 (±0.34) a	0.74 (±0.27) a	0.80 (±0.23)	a	0.82 (±0.37)	a	1.22 (±0.28)	b	0.69 (±0.59)	a
		RB			0.35 (±0.09)		0.51 (±0.20)		1.01 (±0.22)		0.75 (±0.18)	
	Saliva	Crystals	0.90 (±0.27) ab	1.06 (±0.18) b	0.41 (±0.20)	a	0.45 (±0.28)	a	1.05 (±0.43)	ab	1.33 (±0.31)	a
		RB			0.53 (±0.21)		0.52 (±0.16)		1.11 (±0.38)		1.44 (±0.27)	
	Tea	Crystals	1.56 (±0.65) a	23.01 (±3.24) a	1.27 (±0.49)	a	22.07 (±4.28)	a	0.95 (±0.26)	b	3.15 (±1.33)	a
		RB			1.15 (±0.41)		17.14 (±2.19)		1.27 (±0.42)		4.86 (±1.12)	
	Wine	Crystals	5.66 (±0.43) a	15.75 (±2.99) a	0.61 (±0.35)	n/a	1.71 (±0.87)	b	5.00 (±0.47)	a	15.84 (±2.25)	a
		RB			2.00 (±0.90)		1.22 (±0.70)		4.02 (±1.09)		13.20 (±2.65)	
CC	Coffee	Crystals	0.98 (±0.25) b	1.97 (±0.67) b	1.26 (±0.40)	a	1.81 (±0.33)	a	1.20 (±0.32)	b	1.08 (±0.42)	b
		RB			1.25 (±0.28)		2.02 (±0.88)		1.13 (±0.59)		0.98 (±0.18)	
	Cola	Crystals	0.88 (±0.32) a	0.87 (±0.26) a	0.89 (±0.37)	a	0.61 (±0.37)	a	0.97 (±0.59)	b	1.13 (±0.41)	a
		RB			0.84 (±0.25)		0.41 (±0.17)		1.31 (±0.18)		0.92 (±0.40)	
	Saliva	Crystals	1.20 (±0.51) a	1.33 (±0.57) b	0.67 (±0.30)	a	0.71 (±0.21)	a	1.17 (±0.45)	a	0.99 (±0.51)	ab
		RB			0.47 (±0.43)		0.71 (±0.49)		1.15 (±0.49)		1.28 (±0.54)	
	Tea	Crystals	0.85 (±0.29) b	19.28 (±3.51) a	0.99 (±0.23)	a	18.87 (±2.45)	a	0.49 (±0.15)	a	1.39 (±0.62)	b
		RB			1.01 (±0.18)		15.71 (±4.49)		0.31 (±0.38)		2.91 (±0.38)	
	Wine	Crystals	1.47 (±0.25) b	1.22 (±0.47) b	2.03 (±0.43)	n/a	1.40 (±0.68)	ab	1.26 (±0.32)	b	1.23 (±0.40)	b
		RB			1.90 (±0.20)		1.13 (±0.48)		1.18 (±0.25)		0.97 (±0.68)	
MTM	Coffee	Crystals	0.91 (±0.32) b	1.45 (±0.42) b	1.32 (±0.39)	a	1.78 (±0.41)	a	0.55 (±0.15)	c	0.61 (±0.18)	c
		RB			1.12 (±0.30)		1.01 (±0.38)		0.79 (±0.32)		0.85 (±0.30)	
	Cola	Crystals	0.65 (±0.33) a	0.78 (±0.21) a	0.59 (±0.29)	a	0.52 (±0.09)	a	0.54 (±0.24)	a	0.86 (±0.18)	a
		RB			0.72 (±0.26)		0.66 (±0.14)		0.94 (±0.28)		0.89 (±0.30)	
	Saliva	Crystals	0.63 (±0.29) b	0.66 (±0.23) a	0.58 (±0.11)	a	0.68 (±0.29)	a	0.68 (±0.18)	b	0.96 (±0.32)	b
		RB			0.55 (±0.13)		0.66 (±0.08)		0.75 (±0.31)		0.70 (±0.19)	
	Tea	Crystals	1.03 (±0.36) b	21.21 (±2.98) a	1.37 (±0.41)	a	21.02 (±2.01)	a	0.81 (±0.50)	b	0.73 (±0.26)	b
		RB			0.86 (±0.38)		19.16 (±3.62)		0.77 (±0.24)		2.14 (±1.33)	
	Wine	Crystals	0.90 (±0.26) c	0.81 (±0.42) b	0.97 (±0.27)	n/a	0.69 (±0.09)	a	0.87 (±0.27)	c	0.90 (±0.29)	b
		RB			1.39 (±0.20)		0.66 (±0.40)		0.79 (±0.30)		0.86 (±0.33)	

*SD* standard deviation, *CC* ClearCorrect, *INV* Invisalign, *MTM* Minor Tooth Movement, *RB* Retainer Brite, *IC* Intergroup comparison. Intergroup comparison of color differences among the various aligner types according to the Tukey’s multiple comparison test. Non-identical letters (a, b and c) indicate a statistically significant difference between aligner types in each solution at each time point (*P* < 0.05). n/a was used as the t-test demonstrated a different effect among the two cleansers as described in the results section

After a seven-day immersion in staining agents, the color changes are enhanced. Again, there is a significant difference in mean values for INV compared to its competitors for coffee (Brunner-Langer, *p* < 0.0001 for INV-CC and INV-MTM) and red wine (Brunner-Langer, *p* < 0.0001 for INV-CC and INV-MTM). A seven-day exposure to tea created high mean values of ΔE ± standard deviation from baseline for the three brands (ΔE INV ± SD = 23.01 ± 3.24; ΔE CC ± SD = 19.28 ± 3.51; ΔE MTM ± SD = 21.21 ± 2.98) without any statistically significant difference between them. (Fig. 2).

**Fig. 3:**
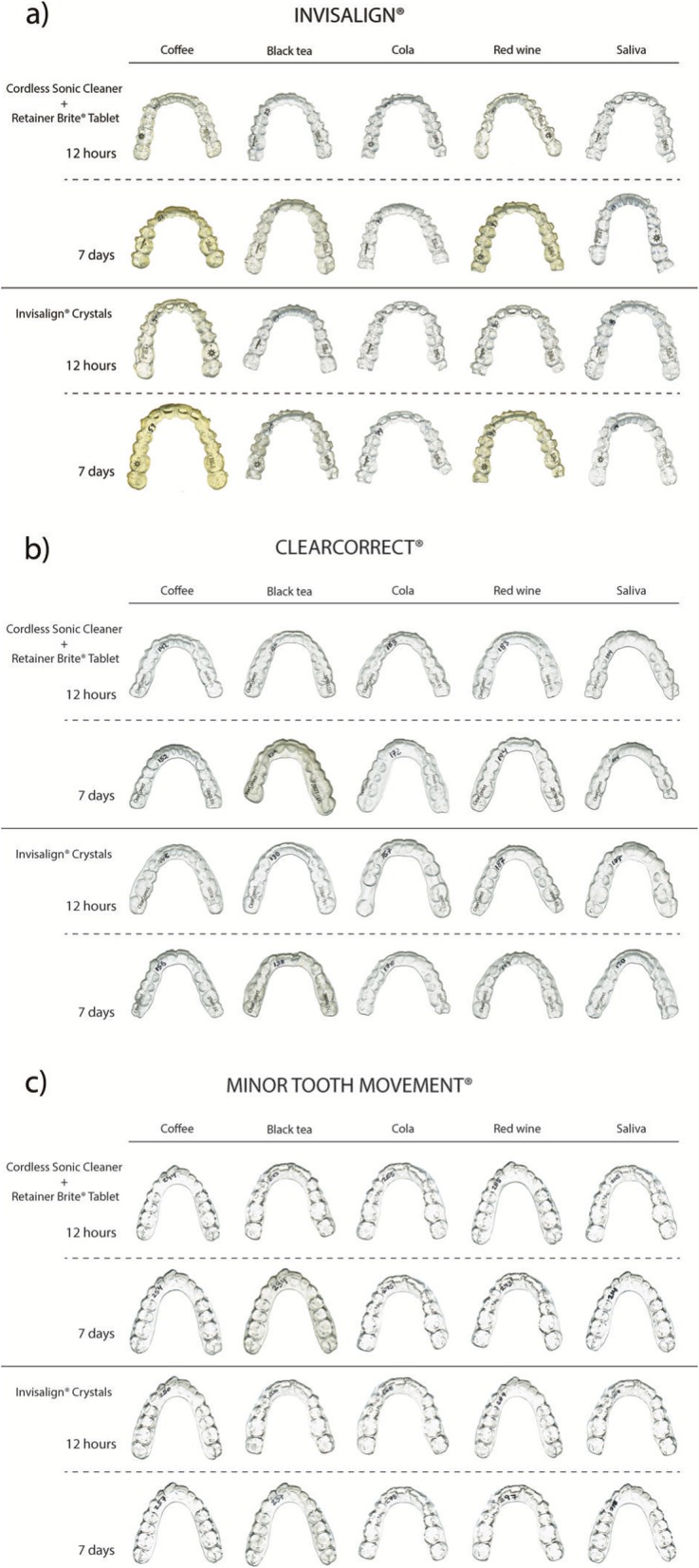
Photographs of the aligners after staining and cleansing cycles by either the Invisalign® crystals or the Retainer Brite® tablet combined with the Cordless Sonic Cleaner (**a**) Invisalign® (**b**) ClearCorrect® (**c**) Minor Tooth Movement®

The comparisons of the mean values of ΔE between T3 and T1 showed that the Retainer Brite® tablet combined with the sonic bath cleaned the INV (t-test, *p* 2-tailed = 0.012) and MTM (t-test, *p* 2-tailed = 0.024) aligners exposed to wine during 12 h significantly more than the INV crystals (Fig. 3).

The comparisons of the mean values of ΔE between T3 and T2 did not demonstrate any interaction between brands and cleansers. However, we observed that the mean values of color change for the cleaning of the aligners exposed 7 days to tea were important (ΔE INV ± SD = 19.60 ± 4.13; ΔE CC ± SD = 17.29 ± 3.80; ΔE MTM ± SD = 20.09 ± 2.93) although there was no statistically significant difference between them (Fig. 3).

The measurements of the color changes between T3 and T0 for the 12-h immersion groups showed that the INV shells exposed to coffee or red wine were significantly more stained even after a cleaning by either technique compared to CC and MTM (coffee: Brunner-Langer, *p* = 0.0010 for INV-CC and *p* < 0.0001 for INV-MTM / wine: Brunner-Langer, *p* < 0.0001 for both INV-CC and INV-MTM) (Fig. 3).

For the 7-day exposure groups (T3-T0), we observed that the INV aligners immersed in coffee or red wine were significantly more stained than the other two brands after a cleansing cycle (Brunner-Langer, *p* < 0.0001 for INV-CC and INV-MTM for both coffee and red wine). The INV aligners were also more stained than CC or MTM after a 7-day exposure to black tea followed by a cleaning, but to a lesser extent. (two-way ANOVA, *p* = 0.001 for INV-CC and *p* < 0.001 for INV-MTM) (Fig. 3).

No relevant statistically significant variation of ∆E was noted between the CC and the MTM aligners for the different time intervals.

Independent samples t-tests were carried out in order to compare the mean values of ∆E at 12 h and at 7 days per solution and per brand. The differential was statistically significant for coffee (INV: *p* 2-tailed < 0.001 / CC: *p* 2-tailed < 0.001 / MTM: *p* 2-tailed = 0.004), tea (*p* 2-tailed < 0.001 for INV, CC and MTM separately) and wine to a lesser extent (INV: *p* 2-tailed < 0.001). Where a statistically significant differential is assessed, we can interpret that a color change continued between 12 h and 7 days.

**Fig. 4:**
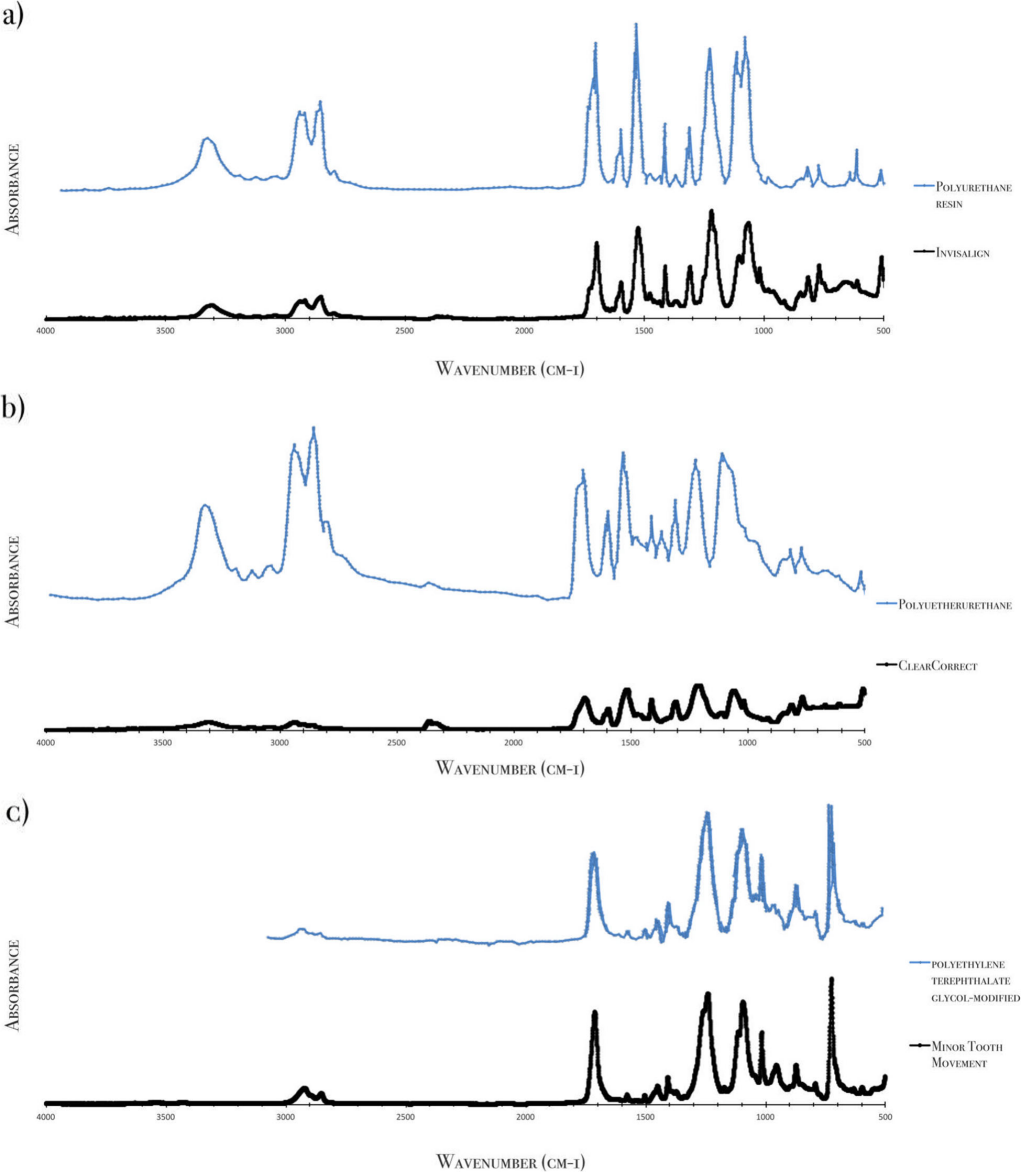
FTIR spectra of the aligner polymers. (**a**) Invisalign® (**b**) ClearCorrect® (**c**) Minor Tooth Movement®. FTIR: Fourier transform infrared spectroscopy

ATR-FTIR confirmed that the INV and CC appliances are made of a polyurethane-based material [21, 22], whereas the MTM aligners are a PETG-based polyester [23]. (Fig. 4). The FTIR spectrum of CC aligners shares features with that of polyetherurethane, but only a 63% match, which may indicate differences in polymer chain lengths, specific tailored functionalities or the presence of additives.

### Limitations

Among the limitations of the study, only one experimenter took the measurements with the Adobe Photoshop® CS6 software for all the aligners, offering no inter-rater reliability to this study. In the same vein, the data gathering of the different CIELAB parameters or the FTIR spectra might have been performed more than once to determine an intra-rater reliability. Moreover, it would have been possible to produce the mean values of the color changes with more than five points per arch, giving more precision to the results. Another detail to consider comes from the fact that, despite the great care taken during measurements, the five pixels used from each image were not necessarily positioned at the same place between T0, T1, T2, T3. The time between aligner removal from the coloring solutions and the onset of the cleaning phase was not rigorously controlled; this might have led to stains harder to remove by the two cleansers. Our study only considers one brand of coloring media among the plurality on the market. As this is an in vitro study, it does not replicate the normal oral conditions with real individuals wearing the appliances during the recommended time. As an intermediate approach, it would have been possible to incorporate some saliva replacement gel within the four coloring media in order to approach in vivo conditions, or to vary temperature.

## Discussion

Polyurethane has interesting features like high elasticity, flexibility, chemical resistance, oxidation resistance, mechanical strength and ease of processing [7, 24]. In a previous study on mechanical and chemical properties of aligners, thermoplastic polyurethane used in the INV devices showed high hardness and elastic modulus but less creep resistance [17]. PETG, used in MTM, demonstrates high wear resistance, transparency, high strength, high dimensional stability and solvent resistance [17, 23]. FTIR analysis of INV aligners shows the following characteristic molecular bands: NH (3307 cm^− 1^), CH (2917 cm^− 1^, 2851 cm^− 1^, 1413 cm^− 1^, 1017 cm^− 1^ and 915 cm^− 1^), aromatic CH (1596 cm^− 1^, 816 cm^− 1^ and 769 cm^− 1^), C=O of NCO (1698 cm^− 1^), C=O (1309 cm^− 1^), N-H and C=O of NCO (1526 cm^− 1^), C-O (1219 cm^− 1^) and C-O-C (1104 cm^− 1^ and 1064 cm^− 1^). The CC spectra have multiple molecular bands in common with those of INV: NH (3305 cm^− 1^), CH (2935 cm^− 1^, 2860 cm^− 1^, 1412 cm^− 1^, 1017 cm^− 1^ and 914 cm^− 1^), aromatic CH (1596 cm^− 1^, 813 cm^− 1^ and 766 cm^− 1^), C=O of NCO (1697 cm^− 1^), C=O (1308 cm^− 1^), N-H and C=O of NCO (1515 cm^− 1^), C-O (1216 cm^− 1^) and C-O-C (1112 cm^− 1^ and 1059 cm^− 1^). For the MTM aligners made of PETG-based material, their molecular bands can be described as: asymmetrical aliphatic CH (2852 cm^− 1^), symmetrical aliphatic CH (2921 cm^− 1^), other aliphatic CH (1407 cm^− 1^, 1016 cm^− 1^ and 725 cm^− 1^), C=O (1712 cm^− 1^), aromatic CH (1504 cm^− 1^) and C-C-O (1241 cm^− 1^ and 1094 cm^− 1^).

To our knowledge, few articles exist on the comparison of the color stability or transparency of thermoplastic orthodontic aligners among different brands on the market [4, 7–9], with no studies conducted on CC or MTM brands.

An earlier study showed significant color changes within the Vivera® retainers by spectrophotometry [9]. Those retention appliances, also manufactured by Align Technology©, are made of polyurethane blended with methylene diphenyl diisocyanate and 1,6-hexanediol [11]. Coffee also caused color changes in those retainers, to a lesser degree, as well as tea and red wine after a seven-day immersion [9]. This study by Zafeiriadis et al. identified that the precision of their spectrophotometric measurements constituted a limitation [9]. Moreover, they did not compare aligners from different companies unlike in Liu et al. [7, 9]. Indeed, the Liu study compared INV aligners with two Chinese brands [7]. However, contrary to our protocol, they employed distilled water as a control and washed their samples in a ultrasonic cleaner after all 12-h or 7-day exposures, likely affecting their colorimetric measurements [7]. Indeed, their NBS values following a 7-day immersion in black tea for their INV aligners were much less important than ours (notwithstanding the fact that the black tea used was different in the two studies) [7]. Indeed, we showed that cleaning cycles (admittedly more aggressive than those used by Liu et al.) definitely removed black tea pigments.

For the interpretation of the clinical data, according to various articles, a ΔE value superior to 3.3 is visually appreciable by a nonskilled person, which means that the color change is unacceptable in the context of aligners worn for aesthetic reasons [25, 26]. If the ΔE value is smaller than 1, it is considered clinically undetectable (values between 1 and 3.3 are deemed clinically acceptable) [25, 26].

We hypothesize that the INV aligners’ surface porosity, combined with the polar nature of polyurethane, can explain their staining susceptibility compared to CC (also polyurethane) and MTM (PETG) aligners. Indeed, untreated polyurethane has been shown to be a natively porous material [27]. Increased water absorption would encourage the penetration of pigments from the external environment into the polymer [7, 27, 28]. At first, the water molecules are linked to the surface of the aligner before being internalized within the plastic [28]. The interactions between water and polyurethane are facilitated by the fact that this material contains polar carbamate groups -NHCOO- that encourage hydrophilic links with pigments from the aqueous solutions [7, 24]. Furthermore, different grades exist among polyurethanes, which could explain the differences between INV and CC. The polyols that are contained in polyurethane are mainly divided into polyethers and polyesters [24]. Ester groups in polyester polyols have an important polarity, promoting the formation of hydrogen bonds, whereas ether groups are more resistant to hydrolysis and contain more flexible segments [24].

Upon cleaning with either the INV crystals or the Retainer Brite tablets, aligners from all three brands that had been exposed to tea for 7 days reverted almost back to their initial color. This indicates that the two techniques have a good stain-removal potential for the staining compounds in tea, as the differences between T3 and T2 could be easily observed by a nonskilled individual.

The INV appliances stained via exposure to coffee or red wine (12 h or 7 days) still presented a marked color change after cleaning by either method. We can definitely affirm that the cleaners have a better stain-removal potential for tea than for other chromogenic agents, such as those found in coffee and red wine.

As our study is in vitro, it does not exactly represent a normal 7-day aging in a real oral environment. Our aligners were not exposed to oral bacteria or enzymes, to functions (chewing, removal, reinsertion) or parafunctions (bruxism) [8, 9]. Moreover, the possibility that different polymers saturate following exposure to staining agents over long times could be studied in order to better understand the obtained results.

One of the main clinical interests in doing this research was to give the practitioners guidelines regarding dietary instructions for their patients during their orthodontic treatment. Staining agents like coffee, tea and red wine should be especially avoided with the INV appliances. Further studies are required to permit continued evaluation of the optical properties of those appliances in vivo, in order to better depict the real environment in which they are utilized.

## Conclusions

Invisalign® appliances are more prone to pigmentation after a 12-hour or a seven-day exposure to coffee or red wine compared to the ClearCorrect® or Minor Tooth Movement® devices. For its part, black tea caused marked extrinsic stains on the surface of the three different brands of aligners, but these could be readily cleaned away. Neither of the two cleaning methods showed a clinically greater stain-removal potential over the other after immersion into staining agents.

## Supplementary information

**Additional file 1.** Experimental Flowchart. Breakdown of the experimental conditions used to analyze the 100 aligners per brand

## Data Availability

The datasets used and/or analyzed during the current study are available from the corresponding author on reasonable request.

## Abbreviations

ATR: Attenuated Total Reflectance
CC: ClearCorrect
CIELAB: Commission Internationale de l’Eclairage
FTIR: Fourier-transform infrared spectroscopy
INV: Invisalign
MTM: Minor Tooth Movement
NBS: National Bureau of Standards
PETG: Polyethylene terephthalate glycol-modified
ΔE: Delta E (color change)
