# Supplementary figures and images for: Colorimetric and spectrophotometric measurements of orthodontic thermoplastic aligners exposed to various staining sources and cleaning methods
Source: Head Face Med. 2020 Feb 18;16:2. doi: 10.1186/s13005-020-00218-2 (PMC7027305; doi:10.1186/s13005-020-00218-2)

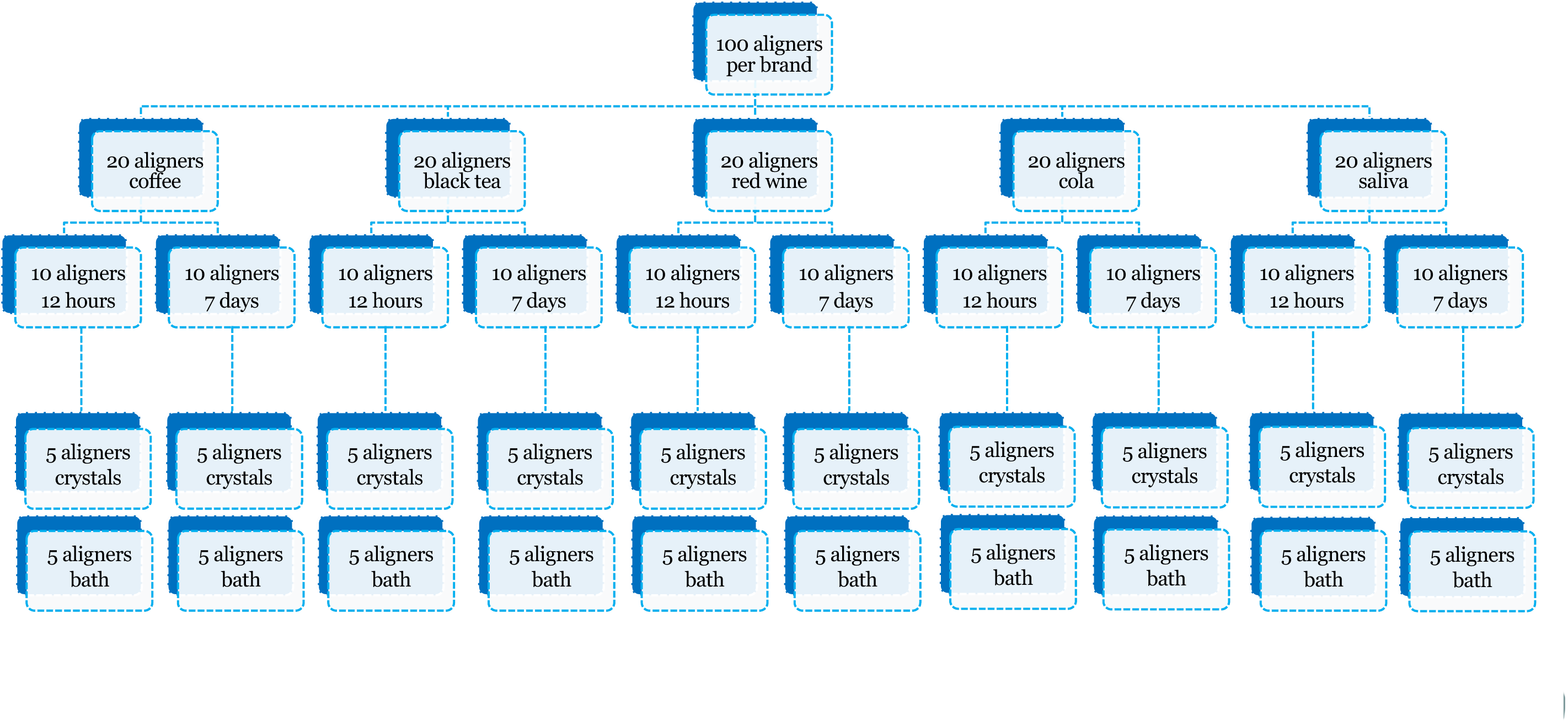

Supplement: Supplementary file 1 — Additional file 1. Experimental Flowchart. Breakdown of the experimental conditions used to analyze the 100 aligners per brand [file 13005_2020_218_MOESM1_ESM.tif]
